# Reductionist modeling of calcium-dependent dynamics in recurrent neural networks

**DOI:** 10.3389/fncom.2025.1565552

**Published:** 2025-06-13

**Authors:** Mustafa Zeki, Tamer Dag

**Affiliations:** College of Engineering and Technology, American University of the Middle East, Egaila, Kuwait

**Keywords:** calcium-activated potassium channels, integrate and fire, neural networks, inhibitory networks, olfactory

## Abstract

Mathematical analysis of biological neural networks, specifically inhibitory networks with all-to-all connections, is challenging due to their complexity and non-linearity. In examining the dynamics of individual neurons, many fast currents are involved solely in spike generation, while slower currents play a significant role in shaping a neuron's behavior. We propose a discrete map approach to analyze the behavior of inhibitory neurons that exhibit bursting modulated by slow calcium currents, leveraging the time-scale differences among neural currents. This discrete map tracks the number of spikes per burst for individual neurons. We compared the map's predictions for the number of spikes per burst and the long-term system behavior to data obtained from the continuous system. Our findings demonstrate that the discrete map can accurately predict the canonical behavioral signatures of bursting performance observed in the continuous system. Specifically, we show that the proposed map a) accounts for the dependence of the number of spikes per burst on initial calcium levels, b) explains the roles of individual currents in shaping the system's behavior, and c) can be explicitly analyzed to determine fixed points and assess their stability.

## 1 Introduction

The primary goal of this study is to characterize the role of calcium-dependent adaptation in bursting neurons observed in many brain regions (e.g., see Lee et al., 2010; Wallén et al., 2007; Matthews et al., 2009; Gold et al., 1996; Behr et al., 1996; Bhattacharjee and Kaczmarek, 2005; Guckenheimer et al., 1993; Bazhenov et al., 2001a). While these models arise in many neuronal systems (e.g., see Traub et al., 2005; Bazhenov et al., 2001a; Moustafa et al., 2008; Zeki and Moustafa, 2017), there has been almost no mathematical analysis of biologically realistic models. This difficulty arises because the models are highly nonlinear, include a large number of parameters, and typically exhibit a complex structure of oscillatory behavior (e.g., see Wilson and Laurent, 2005; Perez-Orive et al., 2004; Bazhenov et al., 2001b; Zeki and Balcı, 2019, 2020).

In our study, we initially examine a network consisting of one excitatory neuron and two inhibitory neurons, where the inhibitory neurons are connected through lateral inhibition and receive synaptic input from the excitatory neuron. As a slow modulatory current, inhibitory cells are assumed to include an intrinsic slow afterhyperpolarization (sAHP) current (e.g., see Lee et al., 2010; Wallén et al., 2007; Matthews et al., 2009; Gold et al., 1996; Behr et al., 1996; Bhattacharjee and Kaczmarek, 2005; Guckenheimer et al., 1993; Bazhenov et al., 2001a). For example, in the case of two inhibitory neurons, one neuron fires and inhibits the other. With ongoing activity, calcium in the active cell increases, which activates the sAHP current. The sAHP current decreases the excitability of the active inhibitory cell. At some point, the second inhibitory cell manages to take over the activity. In this network, we aim to determine the number of spikes each active cell makes per burst [number of spikes per burst (NSPB)] and how it depends on important model parameters.

Our approach for studying a general class of networks is to first reduce the full system of differential equations to a discrete-time dynamical system using a Poincaré map-like approach. This is done systematically so that every parameter in the full model corresponds to some parameter, or combination of parameters, in the discrete model. The discrete model has many advantages over the full continuous model; it is considerably easier to solve numerically, allowing for a more systematic study of how the model's behavior depends on parameters. Moreover, in many cases, we can mathematically analyze the discrete model, which is typically impossible for the full system of equations, except in very special circumstances (see Liu et al., 2015; Terman et al., 1998; Rubin and Terman, 2000 for examples of mathematical analysis at the single neuron level and on networks with relaxation oscillator neurons, respectively).

We reduce the full model to a discrete one by constructing a map, which keeps track of the active and silent (inhibited) cells. That is, if we know which inhibitory cell is active and for how long it has been active, then the discrete map determines which cell fires during the next bursting period. However, it turns out that it is not enough to simply keep track of active and silent inhibitory cells. We must also know what the calcium levels of these cells are. In some sense, the calcium levels can be thought of as a slow variable in the sense of geometric singular perturbation theory (e.g., see Ermentrout and Terman, 2010). If we know which cell is active during a bursting episode and what the initial calcium levels of all the cells, and then the map determines the current number of spikes per burst and which cell is going to be active during the next bursting period.

Transient synchronization phenomena, observed prominently in biological neuronal networks such as the olfactory systems of insects and mammals, play a crucial role in sensory information processing and neural coding. Bazhenov et al. (2001a) and Bazhenov et al. (2001b), through extensive experimental recordings and computational modeling of the locust antennal lobe, explicitly showed that transient synchronization emerges from inhibitory neuronal competition mediated predominantly by calcium-dependent potassium currents. Despite the biological clarity provided by such experimental and computational studies, rigorous analytical frameworks explicitly capturing and predicting these transient synchronization behaviors remain underdeveloped. Our study explicitly addresses this gap by deriving analytically tractable discrete maps of calcium-dependent potassium currents, providing explicit formulas for the number of spikes per burst (NSPB). Such analytical maps allow systematic analysis of stability, bifurcations, and parameter sensitivity, significantly deepening theoretical insights into transient synchronization and directly complementing the foundational work by Bazhenov et al. (2001a) and Bazhenov et al. (2001b).

We begin this study by considering small excitatory-inhibitory networks with special network architectures. By considering small networks, we are able to more easily demonstrate how the discrete map is constructed. Moreover, we perform a detailed mathematical analysis of the types of solutions that these networks exhibit and how they depend on parameters. The analysis leads to concrete formulas for the number of spikes each cell exhibits during each episode. In particular, we obtained an explicit formula for the number of spikes per burst, depending on initial calcium values and system parameters (see Lemma 3.4). Using this formula, we constructed an explicit map and analyzed the existence and stability of its fixed points for various networks (see Theorem 8). The discrete map leads to a clear understanding of the roles each component of the model, including the ionic currents, plays in generating the network behavior.

## 2 Materials and methods

Inhibitory cells (IC) were modeled as a single compartment with currents that are governed by integrate-and-fire kinetics as follows:


(1) $$\begin{array}{l} C_{m}\frac{dv}{dt}=-\left(g_{l}\left(v-E_{L}\right)+I_{AHP}\right)+I_{syn}+I_{app} \end{array}$$

where *C*_*m*_ is the membrane capacitance, which has a value of 1μ*F*, *g*_*l*_ is the leakage conductance with *g*_*l*_ = 0.18μ*S*, *E*_*L*_ is the leak reversal potential with *E*_*L*_ = −60*mV*, and *I*_*syn*_ denotes the sum of synaptic currents. In this study, *I*_*AHP*_ is *Ca*^2+^ dependent *K*^+^ current (Huguet et al., 2016; Sloper and Powell, 1979; Bazhenov et al., 2001a,b; Terman et al., 2002). The applied current *I*_*app*_ is set to a value of 0.2*nS*.

Cells are assumed to fire an action potential when the membrane potential reaches a threshold level *v*_*T*_ = −50*mV*. Once the threshold level is crossed, the membrane potential is reset to the level *v*_*R*_ = −75*mV*.

The intrinsic *I*_*AHP*_ current is described by the following:


(2) $$\begin{array}{l} I_{AHP}=g_{AHP}\left(v-E_{K}\right)\frac{\left[Ca\right]}{\left[Ca\right]+k_{1}} \end{array}$$

with maximal conductance *g*_*AHP*_ = 50μ*S*. *I*_*AHP*_ rate constant *k*_1_ = 10. The reversal potential for *I*_*AHP*_ is *E*_*K*_ = −90*mV*. The calcium concentration [*Ca*] is assumed to increase by 1 μ*M* with every action potential and decays exponentially according to the equation


(3) $$\begin{array}{l} {\left[Ca\right]}^{\prime }=-k_{ca}\left[Ca\right] \end{array}$$

with the decay rate parameter $k_{Ca}=0.001ms^{-1}$.

The calcium dynamics used in this model follow an exponential decay form that reflects the experimentally observed behavior of calcium-dependent potassium currents in hippocampal neurons, as reported by Sloper and Powell (1979). This modeling approach has also been employed in several biologically detailed neuronal models (Traub et al., 2005; Bazhenov et al., 2001a,b). While more complex calcium handling mechanisms exist, including buffering and multi-compartmental effects, the effective decay rate used here serves as a biologically grounded approximation that enables analytical tractability without sacrificing key dynamical features relevant to the bursting behavior and inhibitory competition under study.

An excitatory cell (EC) is modeled as follows:


(4) $$\begin{array}{l} C_{m}\frac{dv}{dt}=-g_{l}\left(v-E_{L}\right)+I_{stim} \end{array}$$

The parameters are identical to those of the inhibitory cell. The applied current for the EC is given as *I*_*stim*_ = 2*nS*.

### 2.1 Synaptic currents

The synaptic inhibition between the inhibitory cells is given as *I*_*syn*_ = *I*_*I*→*I*_+*I*_*E*→*I*_. The currents *I*_*I*→*I*_ and *I*_*E*→*I*_ are modeled as


$$I_{I\to I}=g_{i}s_{i}\left(v-E_{GABA}\right)$$

$$I_{E\to I}=g_{e}s_{e}\left(v-E_{AMPA}\right)$$

with peak synaptic conductance *g*_*i*_ = 25μ*S* and *g*_*e*_ = 4μ*S*, respectively. The synaptic activation variables *s*_*i*_ and *s*_*e*_ are reset to 1 with every action potential and decay exponentially according to the equations


$$s_{i}^{\prime }=-\beta_{i}s_{i}$$

and


$$s_{e}^{\prime }=-\beta_{e}s_{e}$$

with decay rates given as $\beta_{i}=0.1ms^{-1}$ and $\beta_{e}=2ms^{-1}$, respectively.

### 2.2 Network geometry

**Figure 1:**
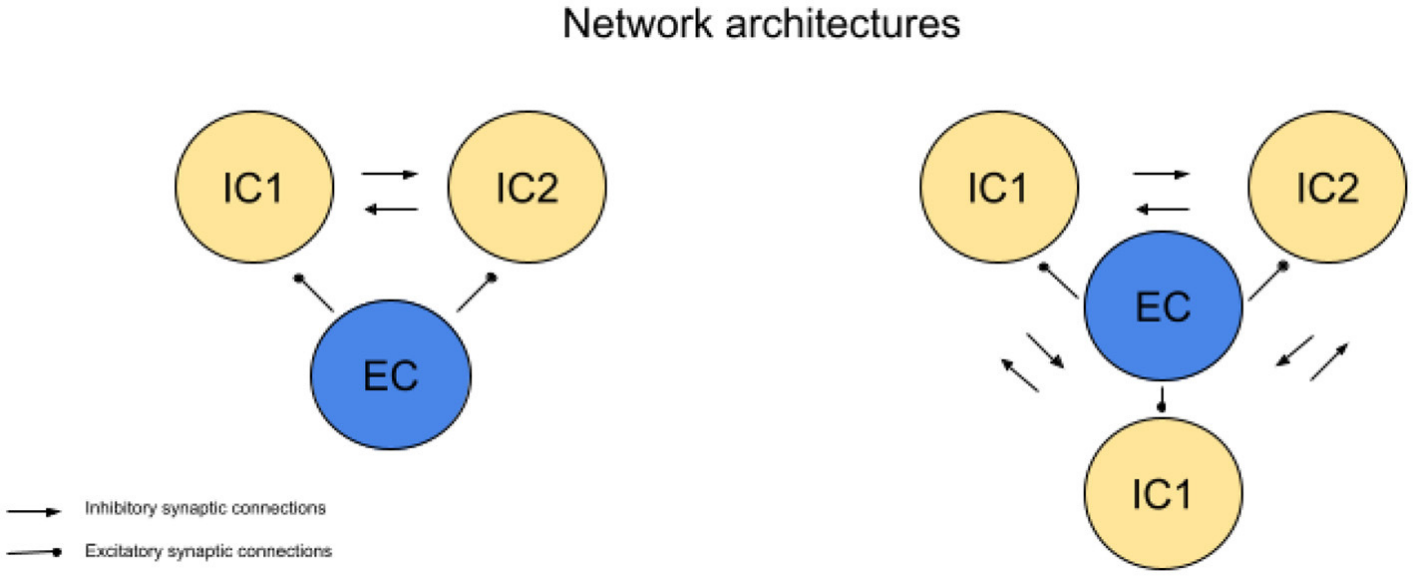
Network geometries. Simulations of networks consisting of 2 inhibitory cells (IC) and one excitatory cell (EC), as well as 3 ICs and 1 EC, are presented. In both configurations, the systems exhibit all-to-all coupling. Regular arrowheads indicate excitatory connections, while oval arrowheads represent inhibitory connections.

We analyze networks consisting of both excitatory and inhibitory neurons, focusing on configurations with one excitatory cell and two or more inhibitory cells (see Figure 1). In these networks, all neurons are connected through all-to-all coupling, allowing for direct interactions between every neuron. This architecture enables competition between inhibitory cells in response to excitatory input, a key aspect we explore in this paper, though the precise mechanism of this competition will be detailed later.

## 3 Results

### 3.1 Simple networks

#### 3.1.1 Network behavior

**Figure 2:**
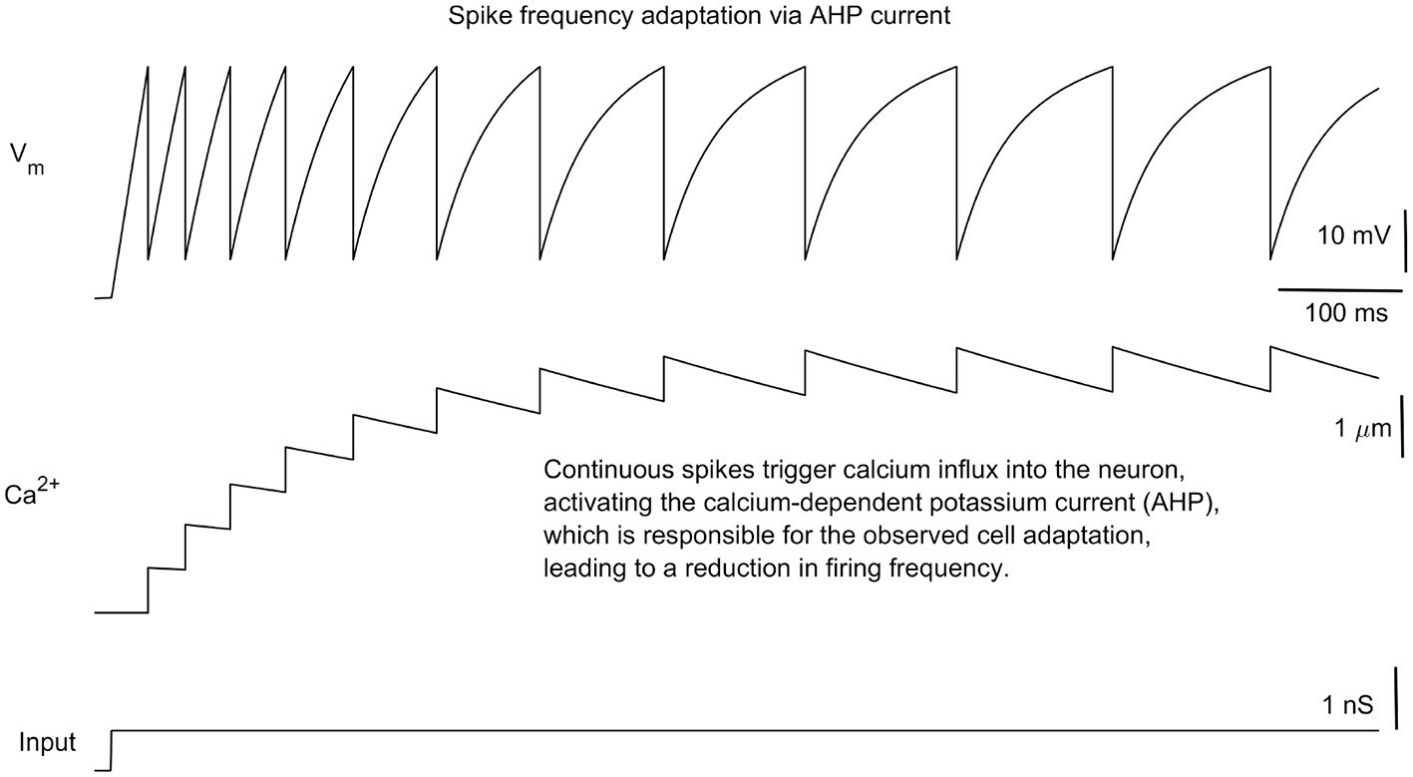
Oscillations in inhibitory cells. The response of the inhibitory cell (IC) to square pulses results in spike frequency adaptation. With each spike, calcium current activation increases, thereby activating the calcium-dependent potassium current. This potassium current has a hyperpolarizing effect, causing the membrane potential to become more negative.

In Figure 2, the behavior of a single IC in response to a step current is displayed together with the [*Ca*] dynamics. In response to the input current, inhibitory cells fire, but inter-spike intervals gradually increase with time. This is due to the AHP current that activates with the build-up of calcium (Figure 2). AHP current is a hyperpolarizing current (making the membrane potential more negative). With continued spiking, interspike intervals reach a point at which the increase in calcium is compensated by its slow decay. Hence, inter-spike intervals eventually stabilize to a constant level.

In a 2IC-1EC network, each inhibitory cell (IC) receives synaptic inhibition from the other (see network architecture, Figure 1). Inhibitory cells compete to spike in response to excitatory input from the excitatory cell (EC). The competition between ICs is primarily driven by the calcium levels within the ICs and the strength and decay rate of the slow inhibitory currents between them.

**Figure 3:**
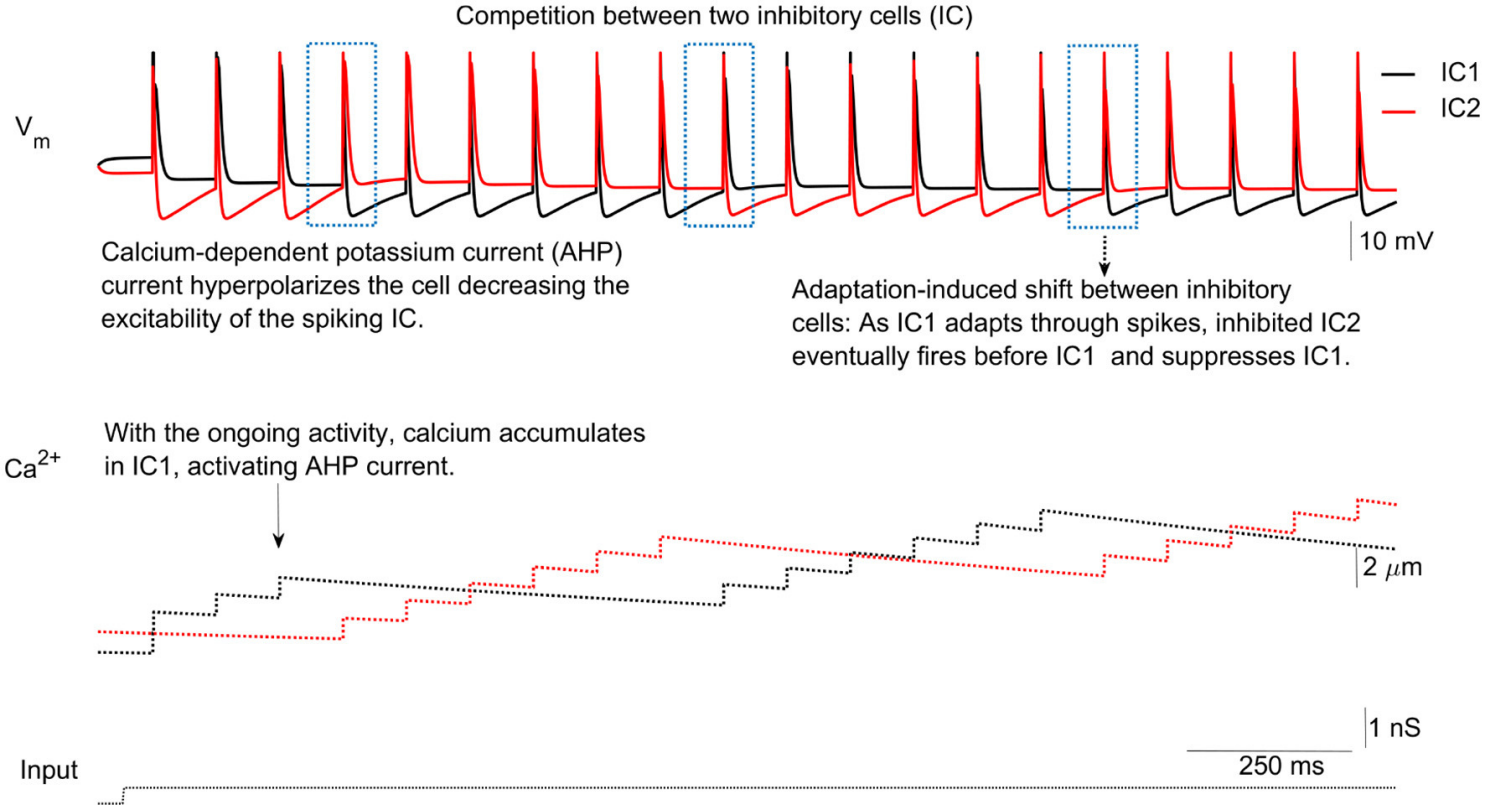
IC competition. In response to synaptic input from the excitatory cell (EC), inhibitory cells (ICs) compete to spike (upper panel). The key factor determining which IC fires is the activation of the calcium-dependent potassium current (AHP), which increases with rising calcium levels (second panel). Prolonged activation of the AHP current makes the membrane potential more negative, decreasing the likelihood of further spiking. This process determines which IC remains active and which becomes silent.

The network functions as follows: When excitatory input is received, one inhibitory cell, called the active IC (aIC), fires an action potential and inhibits the other, referred to as the silent IC (sIC), via inhibitory-to-inhibitory (I-to-I) slow inhibition (see Figure 3). The aIC may continue spiking for several cycles, maintaining inhibition of the sIC. However, with each spike, calcium builds up in the aIC, activating the calcium-dependent potassium current (*I*_*AHP*_), which gradually decreases the excitability of the aIC (see Figure 3, second panel).

The sIC eventually becomes active after a few cycles, depending on the kinetics and strength of the *I*_*AHP*_ and *I*_*I*→*I*_ currents, and takes over the spiking activity. This allows the roles of the ICs to switch. During the subsequent burst, calcium levels in the former aIC decay while they increase in the newly active sIC (see Figure 3). Through this process, ICs alternate in firing bursts in response to the excitatory input.

**Figure 4:**
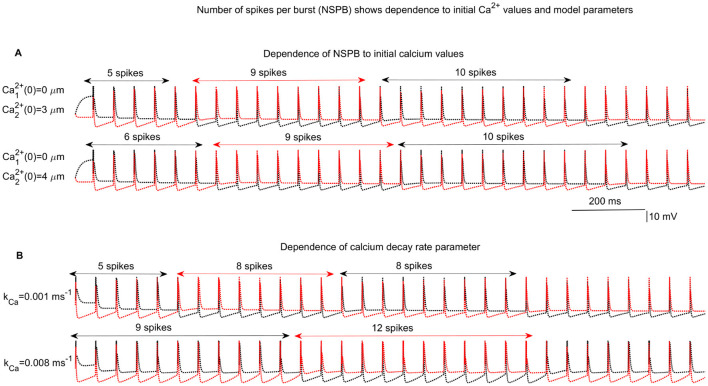
Example network behaviors. Competition among inhibitory cells (ICs) in a 2IC-1EC network results in varying numbers of spikes per burst (NSPB) depending on the initial calcium levels of the ICs and other key network parameters. **(A) Upper**: Comparison of two cases with initial calcium concentrations of $Ca_{1}^{2+}\left(0\right)=0\;mM$ and $Ca_{2}^{2+}\left(0\right)=3mM$ vs. $Ca_{1}^{2+}\left(0\right)=0\;mM$ and $Ca_{2}^{2+}\left(0\right)=4\;mM$, both with a calcium decay rate of $k_{Ca}=0.001\;{\text{ms}}^{-1}$. **(B)** In the second simulation, *k*_*Ca*_ is varied: $k_{Ca}=0.001\;{\text{ms}}^{-1}$ vs. $k_{Ca}=0.008\;{\text{ms}}^{-1}$ with initial calcium values of $Ca_{1}^{2+}\left(0\right)=0\;mM$ and $Ca_{2}^{2+}\left(0\right)=3\;mM$ for IC1 and IC2, respectively.

The number of cycles required for the interchange between the active IC (aIC) and the silent IC (sIC), which corresponds to the number of spikes per burst (NSPB), varies depending on the kinetic and conductance parameters, as well as the initial values of the activation variables for the *I*_*AHP*_ and *I*_*I*→*I*_ currents (Figure 4). In Figures 4A, B, different initial conditions for *Ca*^2+^ concentrations and varying values of the *Ca*^2+^ decay parameter (*k*_*Ca*_) lead to different NSPB outcomes. The NSPB does not stabilize immediately and shows variability from burst to burst. One of the primary objectives of this study is to characterize the evolution of NSPB and explore its variation based on initial *Ca*^2+^ levels and other key model parameters.

### 3.2 Approximation of pre-excitation potential values

At the subthreshold levels, the only active currents are *I*_*AHP*_, *I*_*L*_, and the synaptic inhibition *I*_*I*→*I*_. The changes in the *I*_*AHP*_ and *I*_*I*→*I*_ are very slow within the inter-spike interval. That is, in the ICs model


$$\frac{d\left[Ca\right]}{dt}≅o\left(\epsilon \right)\;\text{and }\frac{ds_{i}}{dt}≅o\left(\epsilon \right).$$

If we let ϵ → 0, then the membrane potential value can be approximated as a fixed point of the potential equation; that is,


$$-\left(I_{l}+I_{AHP}+I_{I\to I}\right)+I_{app}=0$$

or


$$-\left(g_{l}\left(v-E_{L}\right)+g_{AHP}x_{Ca}\left(v-E_{K}\right)+g_{i}s_{i}\left(v-E_{GABA}\right)\right)+I_{app}=0$$

where $x_{Ca}:=\frac{\left[Ca\right]}{\left[Ca\right]+k_{1}}$ (see Equation 2). Solving for *v*, we obtain *v* as a function of [*Ca*]


(5) $$\begin{array}{l} v_{sub}\left(\left[Ca\right]\right):=E_{K}+\frac{g_{l}\left(E_{L}-E_{K}\right)+g_{i}s_{i}\left(E_{GABA}-E_{K}\right)+I_{app}}{g_{l}+g_{AHP}x_{Ca}+g_{i}s_{i}} \end{array}$$

If we can estimate [*Ca*] and *s*_*i*_ amounts of the inhibitory cells at the end of inter-spike intervals, we can approximate preburst subthreshold potential values using the above formula.

In Equation 5, the synaptic inhibition variable *s*_*i*_ decays exponentially from its initial value of 1, with decay rate β_*i*_. Thus, *s*_*i*_ can be approximated explicitly as $s_{i}=e^{-\beta_{i}t_{3}}$, where *t*_3_ is the duration of the inter-spike interval. This demonstrates explicitly how the duration of inhibition influences NSPB calculations.

### 3.3 Calcium dynamics

The concentration of calcium, denoted as [Ca], is assumed to undergo an immediate surge when the membrane potential attains a predetermined threshold level, *v*_*tresh*_. Subsequently, it experiences exponential decay throughout the inter-spike intervals (see Equation 3).

Specifically, for an active inhibitory cell, the calcium dynamics can be represented by the following differential equation:


$$\frac{d[Ca]}{dt}=\{\begin{array}{ll} I_{C}, & \text{if }v=v_{tresh}\\ -k_{Ca}[Ca] & \text{otherwise } \end{array}$$

Here, the constant *I*_*C*_ represents the instantaneous activation of the calcium current. Solving this differential equation explicitly allows us to determine the calcium value at the conclusion of the inter-spike interval based on the initial calcium value. Let *a*_0_ and *s*_0_ represent the calcium values of the spiking (active) and inhibited (silent) inhibitory cells right at the onset of the inter-spike interval, i.e., just before the membrane potential reaches the firing threshold, *v*_*tresh*_. Consequently, the calcium level of the active cell, denoted as [*Ca*]_*a*_, undergoes exponential decay according to the equation derived from the initial value of *a*_0_+*I*_*C*_. Thus, at the conclusion of an inter-spike interval, we obtain the following:


$${\left[Ca\right]}_{a}=\left(a_{0}+I_{C}\right)e^{-k_{Ca}t_{3}}$$

Here, *t*_3_ represents the duration of the inter-spike interval.

Similarly, the calcium level of the inhibited (silent) cell, denoted as [*Ca*]_*s*_, experiences exponential decay according to the same equation. Hence, at the conclusion of the inter-spike interval, we have the following:


$${\left[Ca\right]}_{s}=s_{0}e^{-k_{Ca}t_{3}}$$

.

Assuming that the spiking inhibitory cell maintains its activity for *k* consecutive spikes, we can compute the calcium levels of both the spiking ([*Ca*]_*a*_) and silent ([*Ca*]_*s*_) inhibitory cells at the conclusion of the *k*^*th*^ inter-spike interval as follows: Let $r=e^{-k_{Ca}t_{3}}$. Thus, at the conclusion of the first inter-spike interval, we have:


$${\left[Ca\right]}_{a}\left(1\right)=a_{0}r+I_{C}r$$

. This value can serve as the initial value for the second inter-spike interval. Consequently, we obtain the following:


$${\left[Ca\right]}_{a}\left(2\right)=\left(a_{0}r+I_{C}r\right)r+I_{C}r=a_{0}r^{2}+I_{C}\left(r^{2}+r\right)$$

At the conclusion of the *k*^*th*^ inter-spike interval, we obtain the following:


$${\left[Ca\right]}_{a}\left(k\right)=a_{0}r^{k}+I_{C}\left(r^{k}+r^{k-1}+...+r\right)=a_{0}r^{k}+I_{C}r\frac{1-r^{k}}{1-r}.$$

By replacing the expression $\frac{I_{C}}{1-r}$ with $\bar{A}$, we derive the following:


(6) $$\begin{array}{l} {\left[Ca\right]}_{a}\left(k\right)=r^{k}a_{0}+\bar{A}\left(1-r^{k}\right) \end{array}$$

Similarly, we determine the calcium level of the inhibited cell ([*Ca*]_*s*_) at the conclusion of the *s*_0_ and the parameter *r* as follows:


(7) $$\begin{array}{l} {\left[Ca\right]}_{s}\left(1\right)=s_{0}r,\;{\left[Ca\right]}_{s}\left(2\right)=s_{0}r^{2},...,\;{\left[Ca\right]}_{s}\left(n\right)=s_{0}r^{n}. \end{array}$$

### 3.4 Explicit formula for number of spikes per burst (NSPB) depending on pre-burst calcium values and on the system constants

Knowing subthreshold [*Ca*] and *s*_*i*_ values alone is not enough to estimate the NSPB; we should, in addition, quantify the effects of [*Ca*] and *s*_*i*_ variables on the excitability of aIC and sIC. In order to reach this goal, we consider the Equation 4 of the potential given in the model. The idea to calculate the NSPB is that the interchange between inhibitory cells will occur right after the subthreshold potential values of the neurons are equal at the end of an inter-spike interval (ISI).

**Figure 5:**
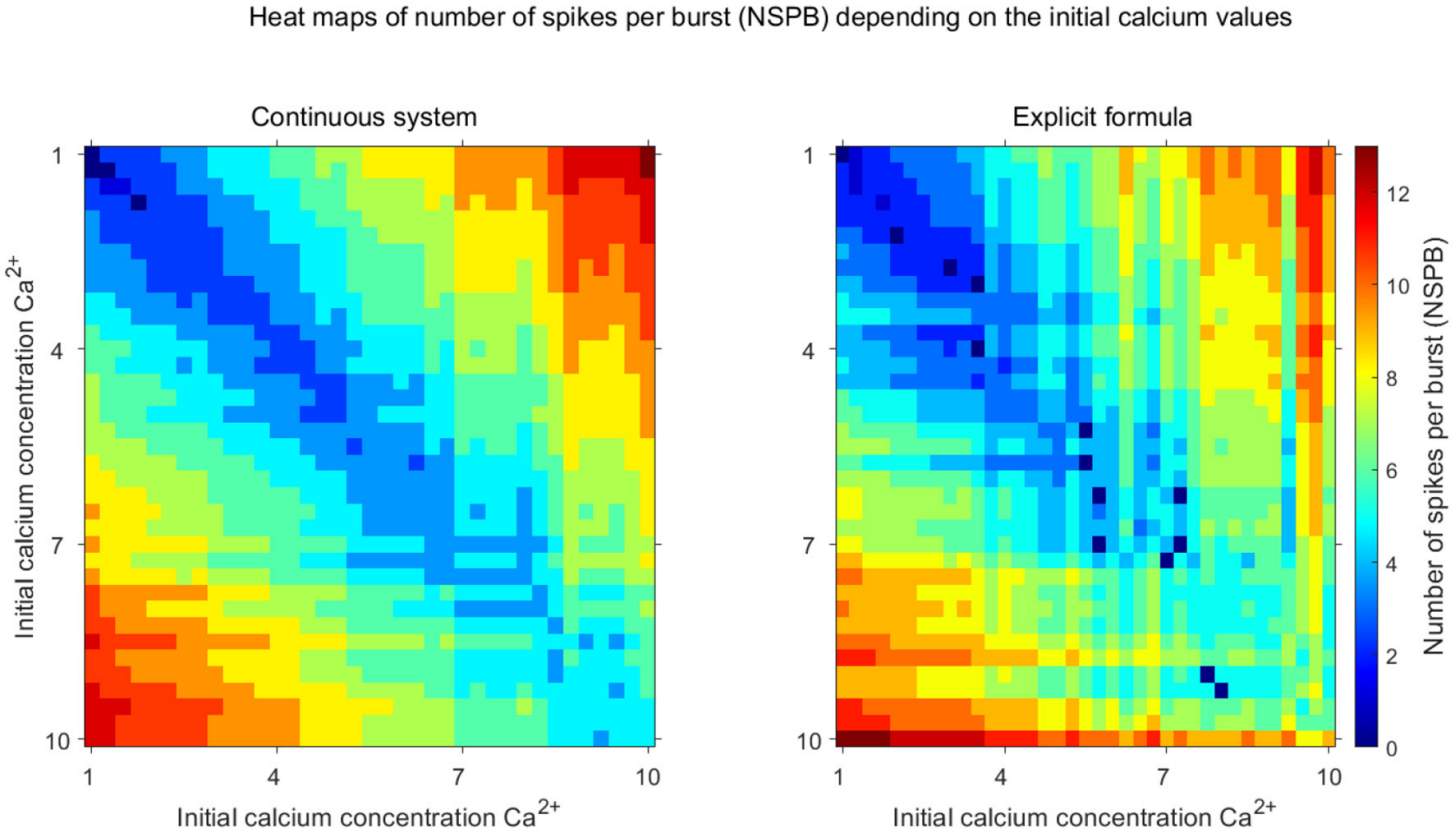
Comparison of the explicit formula with the full system. Heat maps illustrate the stable number of spikes per burst (NSPB) as a function of initial calcium concentrations ([*Ca*^2+^]) for the continuous system (**left**) and the discrete map using the explicit formula (**right**). The color gradient indicates the number of spikes per burst, with warmer colors (red, orange) corresponding to higher NSPB values and cooler colors (blue, cyan) representing lower NSPB values. The axes represent the initial calcium concentrations for inhibitory neurons. These visualizations demonstrate the accuracy of the discrete map in capturing the complex dynamics observed in the continuous model.

Now, combining the ISI-end [*Ca*] and *s*_*i*_ calculation and the quantification of their effects on the ISI-end potential value, we can estimate how many cycles it takes for the excitability of the sIC to be more than that of the aIC, that is, NSPB. If we observe the excitability in terms of the ISI-end potential values, we should calculate how many cycles calcium should accumulate in the aIC so that the ISI-end potential of the aIC would be equal to or less than that of the sIC, as in Figure 3. By combining nth-calcium iteration and potential approximations, we obtained an explicit formula (see Equation 8) for the NSPB of the aIC depending on the initial [*Ca*] values and on the other system parameters and compared NSPBs of the continuous model and explicit NSPB formula, corresponding to different initial [*Ca*] values (see Figure 5C).

We define one burst as a combination of cycles until the interchange between aIC and sIC occurs. To understand when exactly this interchange occurs, we look at the subthreshold potential behavior of the ICs. With the accumulation of [*Ca*], the potential level of aIC is reduced via the *I*_*AHP*_ current. The interchange will occur right after the potential level of the aIC is equal to that of the sIC (Figure 3). Since we have explicit formulas for the potential level depending on pre-excitation [*Ca*] values of the aIC and sIC after *n* consecutive cycles, we can combine these to obtain an equation for NSPB in terms of other system parameters and ISI-end [*Ca*] values.

**Lemma**. *Let *x*_0_ and *y*_0_ denote the burst-initial* [Ca] *amounts of aIC and sIC, respectively. Then, the number of spikes per burst, that is, NSPB, can be given by*


(8) $$\begin{array}{l} n_{xy}\left(x_{0},y_{0}\right):=ceil\left(n_{Ca}\left(x_{0},y_{0}\right)\right) \end{array}$$

with


(9) $$\begin{array}{l} n_{Ca}\left(x_{0},y_{0}\right)=\frac{\ln\left(\frac{-m_{2}+\sqrt{m_{2}^{2}-4m_{1}m_{3}}}{2m_{1}}\right)}{\ln r} \end{array}$$

where for *x* ∈ ℝ *ceil*(*x*): = min{*z* ∈ ℤ:*z* > *x*}. Here *m*_1_, *m*_2_, *m*_3_ are functions of *x*_0_, *y*_0_ and system parameters.

*Proof*. Let *v*_*a*_(*n*): = *v*_*sub*_([*Ca*]_*a*_(*n*)) and *v*_*s*_(*n*): = *v*_*sub*_([*Ca*]_*s*_(*n*)). Also define *x*_0_: = [*Ca*]_*a*_(0) and *y*_0_: = [*Ca*]_*s*_(0). Assuming the interchange between the active and silent inhibitory cells will occur right after when *v*_*a*_(*n*) = *v*_*s*_(*n*) and using the Equation 5, we obtain


(10) $$\begin{array}{l} \frac{g_{l}\left(E_{L}-E_{K}\right)+I_{app}}{g_{l}+g_{AHP}x_{n}}=\frac{g_{l}\left(E_{L}-E_{K}\right)+g_{i}s_{i}\left(E_{GABA}-E_{K}\right)+I_{app}}{g_{l}+g_{AHP}y_{n}+g_{i}s_{i}} \end{array}$$

with


(11) $$\begin{array}{l} x_{n}:=\frac{{\left[Ca\right]}_{a}\left(n\right)}{{\left[Ca\right]}_{a}\left(n\right)+k_{1}} \end{array}$$

(12) $$\begin{array}{l} y_{n}:=\frac{{\left[Ca\right]}_{s}\left(n\right)}{{\left[Ca\right]}_{s}\left(n\right)+k_{1}} \end{array}$$

(see Equation 2). This equation can be solved for *n* explicitly to obtain the desired formula.

Inverting both sides of the Equation 10, we obtain the following:


(13) $$\begin{array}{l} \frac{g_{l}+g_{AHP}x_{n}}{g_{l}\left(E_{L}-E_{K}\right)+I_{app}}=\frac{g_{l}+g_{AHP}y_{n}+g_{i}s_{i}}{g_{l}\left(E_{L}-E_{K}\right)+g_{i}s_{i}\left(E_{GABA}-E_{K}\right)+I_{app}}. \end{array}$$

Let us introduce the following parameters.


$$\begin{array}{ll} a & =\frac{g_{l}}{g_{l}\left(E_{L}-E_{K}\right)+I_{app}}\\ b & =\frac{g_{AHP}}{g_{l}\left(E_{L}-E_{K}\right)+I_{app}}\\ c & =\frac{g_{l}+g_{i}s_{i}}{g_{l}\left(E_{L}-E_{K}\right)+g_{i}s_{i}\left(E_{GABA}-E_{K}\right)+I_{app}}\\ d & =\frac{g_{AHP}}{g_{l}\left(E_{L}-E_{K}\right)+g_{i}s_{i}\left(E_{GABA}-E_{K}\right)+I_{app}}. \end{array}$$

Substituting the above variables in the Equation 13 we obtain the following simple form.


$$\begin{array}{l} a+bx_{n}=c+dy_{n} \end{array}$$

After substituting *x*_*n*_ and *y*_*n*_ from the Equations 11, 12 into this equation, we obtain the following equation:


(14) $$\begin{array}{l} a+b\frac{{\left[Ca\right]}_{a}\left(n\right)}{{\left[Ca\right]}_{a}\left(n\right)+k_{1}}=c+d\frac{{\left[Ca\right]}_{s}\left(n\right)}{{\left[Ca\right]}_{s}\left(n\right)+k_{1}} \end{array}$$

The Equation 14 can be rewritten as follows:


$$\begin{array}{l} a+b\left(1-\frac{k_{1}}{{\left[Ca\right]}_{a}\left(n\right)+k_{1}}\right)=c+d\left(1-\frac{k_{1}}{{\left[Ca\right]}_{s}\left(n\right)+k_{1}}\right) \end{array}$$

Rearranging this equation, we obtain the following equation:


(15) $$\begin{array}{l} \frac{-b}{{\left[Ca\right]}_{a}\left(n\right)+k_{1}}+\frac{d}{{\left[Ca\right]}_{s}\left(n\right)+k_{1}}=\frac{-a-b+c+d}{k_{1}} \end{array}$$

We previously calculated the formulas for the calcium levels of the spiking and inhibited neurons after *n* spikes (see Equations 6, 7, respectively). Substituting the formulas for [*Ca*]_*a*_(*n*) and [*Ca*]_*s*_(*n*) into the Equation 15 and defining $m:=\frac{-a-b+c+d}{k_{1}}$, we obtain the following equation:


$$\begin{array}{l} \frac{-b}{r^{n}a_{0}+\bar{A}\left(1-r^{n}\right)+k_{1}}+\frac{d}{s_{0}r^{n}+k_{1}}=m \end{array}$$

This equation can be rewritten as follows:


$$\begin{array}{l} \frac{-b}{\left(a_{0}-\bar{A}\right)r^{n}+\bar{A}+k_{1}}+\frac{d}{s_{0}r^{n}+k_{1}}=m \end{array}$$

Combining the fractions, we obtain the following equation:


(16) $$\begin{array}{l} \frac{\left[d\left(a_{0}-\bar{A}\right)-bs_{0}\right]r^{n}-bk_{1}+d\left(\bar{A}+k_{1}\right)}{s_{0}\left(a_{0}-\bar{A}\right)r^{2n}+\left[s_{0}\left(\bar{A}+k_{1}\right)+k_{1}\left(a_{0}-\bar{A}\right)\right]r^{n}+k_{1}\left(\bar{A}+k_{1}\right)}=m \end{array}$$

Rearranging the Equation 16, we obtain the following equation:


(17) $$\begin{array}{l} \;ms_{0}\left(a_{0}-\bar{A}\right)r^{2n}+\\ \;\left(m\left[s_{0}\left(\bar{A}+k_{1}\right)+k_{1}\left(a_{0}-\bar{A}\right)\right]-\left[d\left(a_{0}-\bar{A}\right)-bs_{0}\right]\right)r^{n}\\ +\left(\bar{A}+k_{1}\right)\left[mk_{1}-d\right]+bk_{1}=0 \end{array}$$

Setting


$$\begin{array}{l} m_{1}=ms_{0}\left(a_{0}-\bar{A}\right)\\ m_{2}=\left(m\left[s_{0}\left(\bar{A}+k_{1}\right)+k_{1}\left(a_{0}-\bar{A}\right)\right]-\left[d\left(a_{0}-\bar{A}\right)-bs_{0}\right]\right)\\ m_{3}=\left(\bar{A}+k_{1}\right)\left[mk_{1}-d\right]+bk_{1} \end{array}$$

the Equation 17 becomes the following


$$\begin{array}{l} m_{1}r^{2n}+m_{2}r^{n}+m_{3}=0 \end{array}$$

This equation is a quadratic equation for the variable *r*^*n*^ and can be solved to have


$$\begin{array}{l} r^{n}=\frac{-m_{2}+\sqrt{m_{2}^{2}-4m_{1}m_{3}}}{2m_{1}} \end{array}$$

As a result, we obtain the following equation:


$$\begin{array}{l} n=\frac{\ln\left(\frac{-m_{2}+\sqrt{m_{2}^{2}-4m_{1}m_{3}}}{2m_{1}}\right)}{\ln r} \end{array}$$

### 3.5 Comparison of explicit “number of spikes per burst (NSPB)” formula with continuous system simulations

In Figure 5, we illustrate how NSPB depends on the initial calcium levels using both the continuous model (left panel) and the discrete formula (right panel) derived in the previous Lemma. Notably, the discrete formula qualitatively captures the relationship between NSPB and the initial [*Ca*] values of the inhibitory cells.

When the difference in initial calcium levels between the inhibitory cells is small, both the discrete formula and the continuous model yield a low NSPB, as expected. In such cases, only a few spikes are needed to reduce the excitability of the active inhibitory cell compared to the silent one. Conversely, when the gap between initial calcium levels increases, more spikes are required to hyperpolarize the active IC (see Figure 5).

**Figure 6:**
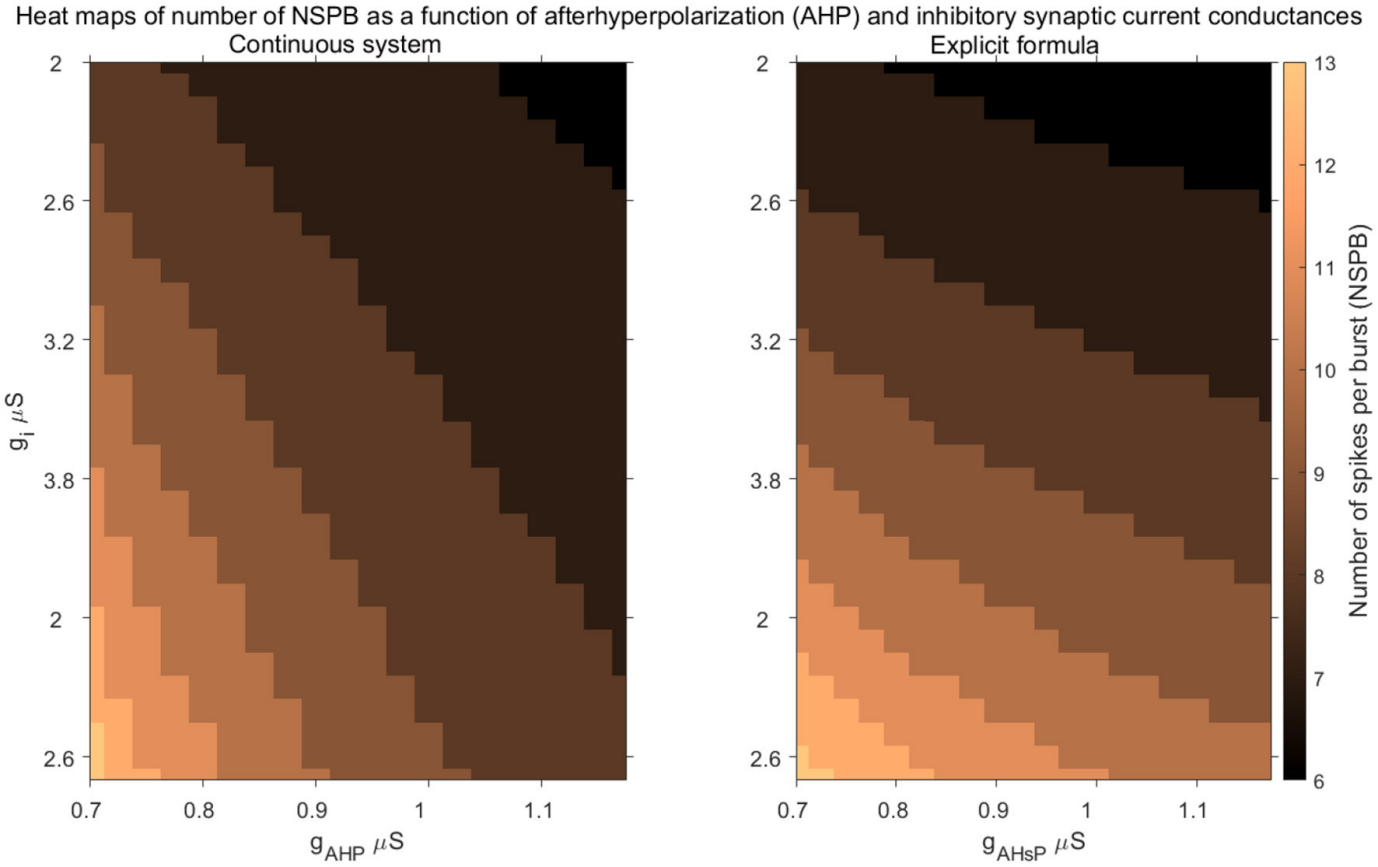
Comparison of the explicit formula with the full system. Heat maps illustrate the stable number of spikes per burst (NSPB) as functions of afterhyperpolarization conductance (*g*_AHP_) and inhibitory synaptic current conductance (*g*_*i*_) for the continuous system (**left**) and the discrete map model (**right**). The color gradient represents the NSPB values, with lighter colors indicating higher NSPB values and darker colors indicating lower values. The figures show how the parameters influence bursting behavior and highlight the strong qualitative correspondence between the continuous model and the discrete map.

In Figure 6, we present heat maps showing the dependence of NSPB on key model parameters, specifically *g*_*AHP*_ and *g*_*i*_, for both the continuous model and the discrete formula. NSPB is inversely related to the conductance of the AHP current, *g*_*AHP*_, because as *g*_*AHP*_ increases, less calcium is needed to hyperpolarize the active IC. On the other hand, NSPB increases with the inhibitory conductance *g*_*i*_, as higher inhibitory conductance requires more calcium to hyperpolarize the active IC, resulting in more spikes per burst.

### 3.6 Discrete map

**Figure 7:**
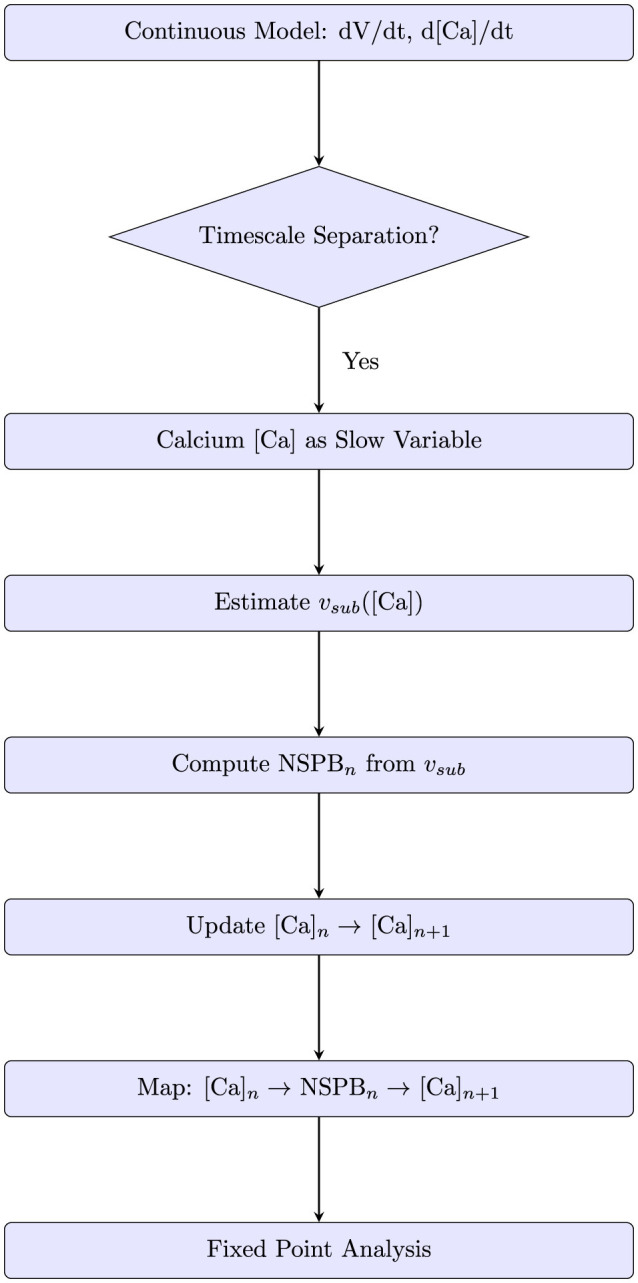
Schematic diagram illustrating the step-by-step derivation of the discrete calcium map from the continuous model. The workflow begins with voltage and calcium dynamics in the continuous system, applies timescale separation, extracts the number of spikes per burst (NSPB), and updates calcium levels analytically.

To clarify the analytical approach used to derive the discrete map from the continuous calcium dynamics model, we provide a detailed schematic diagram (see Figure 7). The diagram illustrates each step involved in constructing the discrete map: starting from the continuous model defined by differential equations for voltage and calcium dynamics, identifying timescale separation to treat calcium as a slow variable, estimating the subthreshold voltage, calculating the number of spikes per burst (NSPB) explicitly, and updating calcium levels accordingly. Finally, this procedure yields an analytically tractable discrete map.

Understanding the NSPB formula allows for the calculation of the initial [*Ca*] values for the next burst. This is achieved by using formulas that estimate the calcium concentrations of inhibitory cells following n-spikes (see Equations 6, 7, respectively). In this manner, a map that calculates the initial calcium values of the (*n*+1)_*th*_ burst based on the *n*_*th*_ burst's initial calcium values and other system parameters is constructed (see Equation 18).

The constructed map is non-linear. However, using certain linearization techniques, the fixed points of the map can be calculated explicitly, and their existence and stability can be established.

To understand the long-term behavior of the network, we examine the following non-linear map by combining the iterated [*Ca*] Equations 7, 6 with the equation for NSPB (Equation 8). Let *C*_*x*_(*k*) and *C*_*y*_(*k*) denote the calcium levels of the aIC and sIC, respectively, at the beginning of the *kth* interchange of the neurons with *k* = 1, 2, .... Let us also define


$$n_{xy}\left(k\right):=n_{xy}\left(C_{x}\left(k\right),C_{y}\left(k\right)\right)$$

as the burst number corresponding to [*Ca*] values *C*_*x*_(*k*) and *C*_*y*_(*k*) (see Equation 8). Therefore, the discrete map to find *k*+1_*th*_ pre-burst calcium values and corresponding NSPB can be set up as follows:


$$\begin{array}{l} C_{x}\left(k+1\right)=r^{n_{xy}\left(k\right)}C_{y}\left(k\right)\\ C_{y}\left(k+1\right)=r^{n_{xy}\left(k\right)}C_{x}\left(k\right)+\bar{A}\left(1-r^{n_{xy}\left(k\right)}\right). \end{array}$$

Let *F*:ℝ^2^ → ℝ^2^ be defined as $F\left(\vec{w}\right)=r^{n_{xy}\left(\vec{w}\right)}D+d\left(\vec{w}\right)$ with, $D:=\left[\begin{array}{cc} 0 & 1\\ 1 & 0 \end{array}\right]$ and $d\left(\vec{w}\right):=\left[\begin{array}{c} 0\\ \bar{A}\left(1-r^{n_{xy}\left(\vec{w}\right)}\right) \end{array}\right]$. Then the discrete map takes the following form:


(18) $$\begin{array}{l} \vec{c}\left(k+1\right)=F\left(\vec{c}\left(k\right)\right)\;\text{with }\vec{c}\left(k\right):=\left[\begin{array}{c} C_{x}\left(k\right)\\ C_{y}\left(k\right) \end{array}\right]. \end{array}$$

Since *r* < 1 with the given parameter set, the following lemma follows.

**Lemma**. *Given* *n* ∈ ℕ^+^, *let* $F_{n}:{\mathbb{R}}^{2}\to {\mathbb{R}}^{2}$ *be defined as* $F_{n}\left(\vec{x}\right)=r^{n}D\vec{x}+{\vec{d}}_{n}$ *with* ${\vec{d}}_{n}=\left[\begin{array}{c} 0\\ \bar{A}\left(1-r^{n}\right) \end{array}\right]$. *Then the map*


(19) $$\begin{array}{l} \vec{x}\left(k+1\right)=F_{n}\left(\vec{x}\left(k\right)\right) \end{array}$$

has stable fixed points $D_{n}:={\left(I_{2}-r^{n}D\right)}^{-1}{\vec{d}}_{n}$.

**Theorem**. Let *D*_*k*_ be defined in the above lemma for *k* ∈ ℕ^+^. If *k*−1 < *n*_*Ca*_(*D*_*k*_) < *k*, then *D*_*k*_ is a stable fixed point of the map (Equation 18).

*Proof*. Since *n*_*xy*_(*D*_*k*_) = *k*, by lemma, *D*_*k*_ is a fixed point of the map (Equation 18). Again by the continuity of the function *n*_*Ca*_, there exist a δ neighborhood *N*_*k*_(δ) of *D*_*k*_, such that *k*−1 < *n*_*Ca*_(*N*_*k*_(δ)) < *k*. ∀ *E*_*k*_ ∈ *N*_*k*_(δ), $||F\left(D_{k}\right)-F\left(E_{k}\right){||}_{2}=r^{k}||D\left(D_{k}-E_{k}\right){||}_{2}\leq r^{k}||D_{k}-E_{k}{||}_{2}<r^{k}\delta$ where ||*x*||_2_ denotes Euclidean norm of *x* ∈ ℝ^2^. This shows that *D*_*k*_ is stable as *r* < 1.

**Corollary**. *Given* *k* ∈ ℕ^+^, *there exists a *g*_*AHP*_ value such that *D*_*k*_ is a fixed point of the map given in Equation 18*.

*Proof*. *n*_*Ca*_(*x*_0_, *y*_0_) given in Equation 9 is a continuous function of the parameter *g*_*AHP*_. For *g*_*AHP*_ ≫ 1, *n*_*Ca*_(*D*_*k*_) < 1 and as *g*_*AHP*_ → 0, *n*_*Ca*_(*D*_*k*_) → ∞; that is, neurons either interchange in the initial cycle or never interchange, respectively. Then, by continuity of *n*_*Ca*_, there exists a *g*_*AHP*_ such that *k*−1 < *n*(*D*_*k*_) < *k*, and hence, *n*_*xy*_(*D*_*k*_) = *k*. By the theorem, *D*_*k*_ is a stable fixed point of the map (Equation 18).

### 3.7 Bifurcation analysis

**Figure 8:**
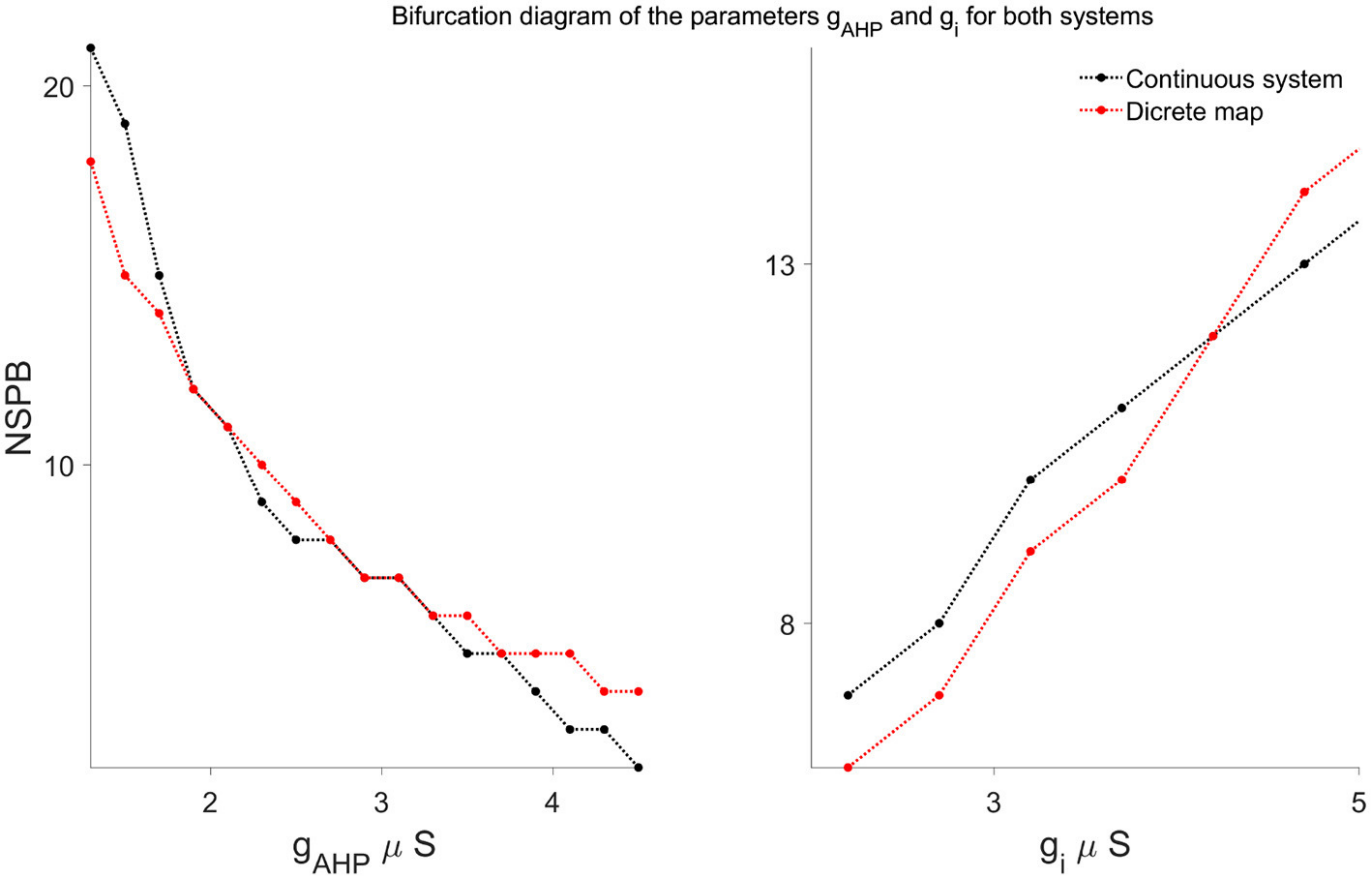
Bifurcation diagrams illustrating the dependence of stable bursting solutions (NSPB) on parameters *g*_AHP_ (**left**) and *g*_*i*_ (**right**), comparing results from the continuous system (black curves) and the discrete map model (red curves). Each point represents the long-term stable number of spikes per burst (NSPB). Both systems exhibit discrete saddle-node bifurcations, where stable NSPB solutions appear or disappear as parameter values vary, demonstrating that the discrete map effectively captures the qualitative dynamical behavior of the continuous model.

To explicitly characterize how parameter changes affect the long-term bursting behavior, we conducted a bifurcation analysis, illustrated clearly in Figure 8. These bifurcation diagrams explicitly show the dependence of the stable number of spikes per burst (NSPB) on critical model parameters, specifically *g*_AHP_ and *g*_*i*_. As these parameters vary, we observe discrete saddle-node (fold) bifurcations, where stable NSPB solutions either appear or disappear, clearly reflecting transitions in the system's qualitative dynamical behavior. These bifurcations are accurately captured by the discrete map, further validating its effectiveness in modeling the system's dynamics.

### 3.8 Generalization to all-to-all couplings

The analysis of small networks is generalized to larger ones. An illustrative example is the nIC-1EC network with all-to-all coupling. Techniques used to analyze the behavior of the 2IC-1EC network are generalized to understand the dynamics of the nIC-1EC network.

The dynamics of the larger network are very similar to 2EC-1EC network: In response to EC excitation, one of the ICs fires and inhibits the other ICs. The firing IC competes with the IC that has the lowest [*Ca*] among the inhibited ICs. The duration of this competition is determined similarly to the 2IC-1EC network.

A discrete map is constructed for this system by ordering ICs with respect to their initial [*Ca*] levels (see Equation 20).

Similar linearization techniques used in the analysis of the 2IC-1EC network are applied to this system to understand the long-term behavior of the system. Here, more interesting solutions, such as alternating NSPBs, were obtained. Through this analysis, explicit formulas for fixed NSPBs and k-periodic (alternating NSPBs) solutions are derived.

We now consider a network of *m*+1 ICs and one EC. All ICs are coupled to all other ICs via inhibition. We order ICs according to their initial calcium amounts as *I*_1_, *I*_2_, ..., *I*_*m*+1_. In response to the EC excitation, *I*_1_, the IC with the lowest calcium level, spikes and inhibits all other ICs. As calcium builds up in *I*_1_, *I*_2_ will be the second IC to spike, as it has the highest level of excitability among all other ICs since it had the second lowest calcium level initially. Here we assume that inhibition is strong enough and calcium builds up fast enough so that the calcium level of the aIC will be more than that of any other sIC after completing its burst. To analyze the network behavior in the long run, we consider the following map.

Let *C*_*x*_(*k*) and *C*_*yi*_(*k*), with *i* = 1, 2, ..., *m* denote calcium levels of aIC and sICs right after the *k*_*th*_ interchange, respectively. Set *n*_*xy*_(*k*) = *n*_*xy*_(*C*_*x*_(*k*), *C*_*y*1_(*k*)), then calcium amounts, and corresponding burst numbers can be followed with the following map:


$$\begin{array}{ll} C_{x}\left(k+1\right) & =r^{n_{xy}\left(k+1\right)}C_{y1}\left(k\right)\\ C_{y1}\left(k+1\right) & =r^{n_{xy}\left(k+1\right)}C_{y2}\left(k\right)\\ .........\\ C_{y\left(m-1\right)}\left(k+1\right) & =r^{n_{xy}\left(k+1\right)}C_{ym}\left(k\right)\\ C_{ym}\left(k+1\right) & =r^{n_{xy}\left(k+1\right)}C_{x}\left(k\right)+\bar{A}\left(1-r^{n_{xy}\left(k+1\right)}\right). \end{array}$$

Define


$${\vec{Ca}}_{m}(k):={\left[\begin{array}{l} C_{x}(k)\\ C_{y1}(k)\\ ...\\ C_{ym}(k) \end{array}\right]}_{(m+1)\times 1},\;{\vec{d}}_{m}(k):={\left[\begin{array}{l} 0\\ ...\\ 0\\ \bar{A}(1-r^{n_{xy}(k+1)}) \end{array}\right]}_{(m+1)\times 1}\text{and}$$

$$\begin{array}{l} E_{m}:={\left[\begin{array}{cccccc} 0 & 1 & 0 & 0 & ... & 0\\ 0 & 0 & 1 & 0 & ... & 0\\ .....\\ 0 & 0 & 0 & ... & 0 & 1\\ 1 & 0 & 0 & ... & 0 & 0 \end{array}\right]}_{\left(m+1\right)\times \left(m+1\right)}. \end{array}$$

Then we obtain following non-linear map,


(20) $$\begin{array}{l} {\vec{Ca}}_{m}\left(k+1\right)=r^{n_{xy}\left(k+1\right)}E_{m}{\vec{Ca}}_{m}\left(k\right)+{\vec{d}}_{m}\left(k\right). \end{array}$$

### 3.9 s-periodic Solutions

Given *s* ∈ ℤ^+^ with *s*<*m*+1 and *l* ∈ ℕ set


$$\begin{array}{l} {\vec{d}}_{l}^{m}:={\left[\begin{array}{c} 0\\ ..\\ 0\\ \bar{A}\left(1-r^{l}\right) \end{array}\right]}_{\left(m+1\right)\times 1}\;\text{and}\\ F_{l}^{m}\left(\vec{x}\right):=r^{l}E_{m}\vec{x}+{\vec{d}}_{l}^{m},\;\vec{x}\in {\mathbb{R}}^{\left(m+1\right)}. \end{array}$$

For a given ${\vec{n}}_{s}:={\left(n_{1},n_{2},...,n_{s}\right)}_{1\times n_{s}}$, we consider the following function of $\vec{x}$


$$\begin{array}{r} F_{{\vec{n}}_{s}}^{m}\left(\vec{x}\right):=F_{n_{s}}^{m}\left(F_{n_{s}-1}^{m}\left(...F_{n_{1}}^{m}\left(\vec{x}\right)\right)\right)\\ =r^{n_{1}+n_{2}+...+n_{s}}E_{m}^{s}\vec{x}+r^{n_{2}+...+n_{s}}E_{m}^{s-1}{\vec{d}}_{n_{1}}^{m}+\\ r^{n3+...+n_{s}}E_{m}^{s-2}{\vec{d}}_{n_{2}}^{m}+...+r^{n_{s}}E_{m}{\vec{d}}_{n_{s-1}}+{\vec{d}}_{n_{s}}. \end{array}$$

If we let ${\vec{d}}_{n_{s}m}:=r^{n_{2}+...+n_{s}}E_{m}^{s-1}{\vec{d}}_{n_{1}}^{m}+r^{n3+...+n_{s}}E_{m}^{s-2}{\vec{d}}_{n_{2}}^{m}+...+r^{n_{s}}E_{m}{\vec{d}}_{n_{s-1}}^{m}+{\vec{d}}_{n_{s}}^{m}$

$F_{{\vec{n}}_{s}}^{m}$ takes the following form:


(21) $$\begin{array}{l} F_{{\vec{n}}_{s}}^{m}\left(\vec{x}\right)=r^{n_{1}+n_{2}+...+n_{s}}E_{m}^{s}\vec{x}+{\vec{d}}_{n_{s}m}. \end{array}$$

Again, since *r* < 1, we obtain the following lemma.

**Lemma**. *Let* ${\vec{n}}_{s},\;m\;\text{and}\;s$ *be given as above. Then the map* ${\vec{x}}_{k+1}=F_{{\vec{n}}_{s}}^{m}\left({\vec{x}}_{k}\right)$ *has stable fixed point* $D_{{\vec{n}}_{s}}$ *given as follows:*


(22) $$\begin{array}{l} D_{{\vec{n}}_{s}}:={\left(I_{m+1}-r^{n_{1}+..+n_{s}}E_{m}^{s}\right)}^{-1}{\vec{d}}_{n_{s}}. \end{array}$$

The following theorem can be proved by the same approach taken to prove the 2IC-1EC case.

**Theorem**. *Let* ${\vec{n}}_{s}=\left(n_{1},..,n_{s}\right)\;\text{and}\;D_{{\vec{n}}_{s}}=\left(v_{1},..,v_{m+1}\right)$ *be defined as above for* *m, s* ∈ ℤ^+^ *with* *s* < *m*. *If* $n_{i}-1<n_{xy}\left(r^{n_{1}+...+n_{i-1}}\left(v_{i},v_{i-1}\right)\right)<n_{i}\;\text{for}\;i=1,2,...\text{s}$, *then* $D_{{\vec{n}}_{s}}$ *is an s-periodic stable point of the map (Equation 20)*.

## 4 Discussion

In this paper, we developed a new, simple discrete model of calcium-modulated bursting inhibitory neurons. The discrete model explains key observations regarding the bursting activity patterns of the cells and is closely related to the singular perturbation approach used to analyze biological neural networks. The model consists of two main parts: 1) a nonlinear function that calculates the NSPB of the active (firing) cell depending on the initial calcium levels, and 2) functions tracking the calcium levels of inhibitory cells (active and silent) depending on the calculated NSPB. The observed bursting activity converged to a constant NSPB, which might change depending on the model parameters. Importantly, the resulting fixed points of the discrete map are stable. Given the nonlinear nature of the resulting map, this is a significant outcome of the study.

The present study focuses on the development and mathematical analysis of a calcium-dependent bursting model derived from biologically grounded principles. While direct validation against calcium imaging or electrophysiological data is beyond the scope of this work, our findings complement previous modeling and experimental studies on transient synchronization in olfactory networks (Bazhenov et al., 2001a,b), where inhibitory competition and calcium-dependent potassium currents play a key role.

Although rebound-like activity is observed in our model, it is important to note that the underlying mechanism does not rely on classical post-inhibitory rebound (PIR) currents, such as T-type calcium (Wang et al., 1991) or h-currents (Lüthi and McCormick, 1998). Rather, the rhythmic switching behavior between inhibitory neurons arises from calcium accumulation during spiking, which activates a calcium-dependent potassium current. This hyperpolarizes the neuron and temporarily suppresses its excitability, thereby allowing its counterpart to become active. Thus, the rebound-like behavior in our system is driven by a calcium-mediated inhibitory competition mechanism, consistent with findings in biologically grounded models of olfactory networks (Bazhenov et al., 2001a,b).

The proposed model exhibited several key properties related to the spiking behavior underlying the bursting dynamics of inhibitory cells. First, the discrete model contained many of the biophysical parameters of the continuous model. Second, the model could replicate the NSPB using the initial calcium values and other important model parameters. Finally, the model enabled explicit mathematical analysis. We demonstrated that the proposed model could explain the alternation between inhibitory cells and the associated NSPB in all-to-all coupled nIC-1EC networks.

Several previous studies have explored calcium-dependent bursting using discrete maps or simulations (e.g., see Channell Jr et al., 2007; Rubin and Terman, 2012; Ibarz et al., 2011). Unlike heuristic or purely numerical methods common in prior work, our key contribution is an explicit closed-form formula (Equation 8) that analytically predicts the number of spikes per burst (NSPB). This explicit formulation enables direct, analytical insights into how calcium levels and key parameters (*g*_AHP_, *g*_*i*_, *k*_ca_) affect burst lengths, facilitating stability analyses and natural extensions to larger networks.

Although we primarily illustrate our findings around a single stable equilibrium, the discrete map derived here indeed supports multiple stable solutions depending on parameters and initial conditions (*g*_AHP_, *g*_*i*_, *k*_ca_, initial calcium concentrations). Our method explicitly allows the exploration of multistability, which we discuss further in Section 4.7 for larger network structures.

We compared the behavior of the model to the data obtained from the continuous system. However, we assumed that the number of excitatory neurons remained constant (one) in the simulated systems. In Terman et al. (2008), the authors developed a discrete map that can function with different numbers of excitatory cells in the network. However, no explicit mathematical analysis for the long-term behavior of the network activity was shown, possibly due to the complications caused by the varying synaptic excitation to the inhibitory cells at each cycle.

In this study, we assumed that the switch between the active inhibitory cell and the silent inhibitory cell occurs when their subthreshold potential levels are equalized. Generalization to random connections can be done by including synaptic excitation to the compared subthreshold potentials. This type of generalization would make sense in the case of instantaneous activation of excitatory synaptic currents (AMPA), as opposed to NMDA-type excitatory synaptic currents that operate on much slower time scales. We obtained promising preliminary results for random network architectures with varying excitatory connections to the inhibitory cells, but we did not include them here to keep the focus of the current study on the bursting duration of the inhibitory cells.

The proposed model not only generated NSPB similar to the continuous system data but also explicitly predicted the long-term NSPB. In summary, our model predicted various aspects of the bursting behavior in an nIC-1EC network with all-to-all couplings in a way that made explicit mathematical analysis of the long-term behavior possible. Our results revealed a high degree of overlap between the discrete map and continuous network results.

## Data Availability

The original contributions presented in the study are included in the article/supplementary material, further inquiries can be directed to the corresponding author.
